# Synthetic Biomaterials for Alveolar Bone Regeneration: A Systematic Review of Clinical Evidence

**DOI:** 10.3390/ma18235328

**Published:** 2025-11-26

**Authors:** Beatrice Bozza, Paolo Pesce, Domenico Baldi, Francesco Bagnasco, Marco Migliorati, Nicola De Angelis

**Affiliations:** Department of Surgical Sciences and Integrated Diagnostics (DISC), University of Genoa, Viale Benedetto XV, 16100 Genova, Italy; s5109128@studenti.unige.it (B.B.); domenico.baldi@unige.it (D.B.); francesco.bagnasco@unige.it (F.B.); or n.deangelis74@gmail.com (N.D.A.)

**Keywords:** biomaterials, alveolar bone regeneration, biphasic calcium phosphate, β-tricalcium phosphate, hydroxyapatite, bioactive glass, bone graft substitutes, dental implants, guided bone regeneration

## Abstract

Background: Alveolar bone resorption remains a major challenge in implant and prosthetic rehabilitation. While autologous bone grafts are still considered the gold standard, their biological and surgical limitations have promoted the use of synthetic biomaterials such as biphasic calcium phosphate (BCP), β-tricalcium phosphate (β-TCP), nanocrystalline hydroxyapatite, and bioactive glass. Methods: This systematic review, conducted in accordance with PRISMA guidelines, was based on a comprehensive search performed in March 2025 across PubMed, MEDLINE, Embase, and Google Scholar. A total of 11 clinical studies—including both randomized and non-randomized comparative trials—were identified. Due to the marked heterogeneity of study designs and outcome measures, meta-analysis was not feasible. Reported outcomes focused on bone volume preservation, residual biomaterial, implant stability, histological integration, and postoperative complications. Results: Overall, synthetic biomaterials achieved satisfactory bone regeneration and implant stability, with mean bone preservation ranging between 85% and 95%, often comparable to xenografts and other grafting materials. Among the materials analyzed, β-TCP and BCP generally demonstrated superior resorption control and dimensional stability, while bioactive glass showed favorable integration and remodeling rates. The addition of bioactive agents such as rhBMP-2, rhPDGF-BB, or platelet-rich plasma further enhanced new bone formation. Conclusions: Within the limits of current evidence, synthetic biomaterials show clinical performance comparable to xenografts, particularly in socket preservation and ridge augmentation procedures. Their predictable handling, absence of donor-site morbidity, and potential for bioactive enhancement make them valuable tools for routine clinical use. Larger, standardized trials with long-term follow-up are needed to validate these findings and refine material selection in alveolar bone regeneration.

## 1. Introduction

Alveolar bone loss represents a frequent and clinically relevant complication in dentistry, arising from trauma, periodontal disease, congenital anomalies, or prolonged tooth absence [1]. This resorption can compromise the functional and esthetic outcomes of prosthetic or implant rehabilitation, emphasizing the need for effective regenerative strategies to restore alveolar integrity.

Autologous bone grafting remains the gold standard due to its osteogenic, osteoinductive, and osteoconductive properties [1]. However, limitations such as donor-site morbidity, limited availability, and increased operative time have encouraged the exploration of alternative grafting materials, including allografts, xenografts, and alloplasts [2,3,4,5].

Bone grafting materials can be broadly categorized into four main groups: autografts (derived from the patient), allografts (from human donors), xenografts (from animal sources), and synthetic alloplasts. Each category presents specific advantages and limitations in terms of biocompatibility, osteoconductivity, and clinical predictability [6,7,8].

Among these, synthetic biomaterials have gained growing attention for their availability, biocompatibility, and tunable physical–chemical properties, which allow customization for specific clinical needs [6,7,8,9]. Their clinical relevance extends beyond performance: synthetic substitutes also overcome ethical and immunological issues associated with human or animal-derived grafts, and they can significantly reduce costs, offering a sustainable and standardized option for bone regeneration [10,11,12,13].

Synthetic materials—such as hydroxyapatite (HA), β-tricalcium phosphate (β-TCP), bioactive glass, and composite ceramics—act as osteoconductive scaffolds that can support bone ingrowth and gradually be replaced by newly formed bone. Their applications include socket preservation, sinus augmentation, ridge reconstruction, and peri-implant defect repair [10,11,12,13]. More recently, advanced biomaterials integrating bioactive ions, polymer-based scaffolds, and nanocomposites have shown promising osteoinductive and angiogenic potential, expanding the scope of synthetic grafting technologies [14,15,16,17].

However, despite encouraging experimental results, clinical evidence remains fragmented and inconsistent. Previous systematic reviews have provided partial insights, often limited by methodological heterogeneity, variable follow-up periods, and inconsistent outcome measures [14,15]. In particular, a lack of standardized protocols and the variability in biomaterial composition hinder the comparison of clinical outcomes across studies [17].

The research gap addressed by this review lies in the need for a unified evaluation of synthetic biomaterials’ clinical performance compared with natural grafts, assessing not only bone volume preservation and implant stability but also long-term integration and complication rates.

The objective of this review is therefore clearly defined: to systematically assess clinical outcomes of synthetic biomaterials used in alveolar ridge preservation and to determine their reliability as alternatives to autologous, allogeneic, or xenogeneic grafts.

This review addresses these gaps by critically evaluating clinical evidence on synthetic biomaterials for alveolar bone preservation. The specific research questions are:

1. Do synthetic biomaterials achieve bone regeneration outcomes comparable to autografts, allografts, and xenografts?
2. How do clinical results vary according to the type of synthetic biomaterial used?
3. What complications, limitations, or methodological biases are reported in the current literature?

By explicitly focusing on synthetic bone substitutes, this review aims to provide clinically meaningful conclusions that may guide decision-making in implant and regenerative dentistry.

## 2. Materials and Methods

Protocol and Registration:

This systematic review was conducted in accordance with the Preferred Reporting Items for Systematic Reviews and Meta-Analyses (PRISMA 2020) guidelines. Quality assessment process followed the PRISMA 2020 Checklist, as detailed in the Supplementary Materials Table S1. A detailed PRISMA flowchart summarizing the study selection process is provided as Supplementary Table S1 to ensure transparency. The protocol was prospectively registered in the PROSPERO database (CRD420251080880).

Eligibility Criteria:

Eligible studies met the following inclusion criteria:

(i). Randomized controlled trials (RCTs) or non-randomized comparative studies with a clearly defined control group;
(ii). Standardized interventions involving synthetic biomaterials for alveolar bone volume regeneration;
(iii). Comparable baseline characteristics among participants;
(iv). Consistent and well-defined outcome measures (e.g., new bone formation, graft resorption, implant survival);
(v). Adequate reporting of quantitative data (means, standard deviations, or confidence intervals) enabling statistical synthesis.

Exclusion criteria included animal or in vitro studies, case reports, narrative reviews, conference abstracts, and studies with insufficient data for extraction.

Population, Intervention, Comparison, Outcomes (PICO):

- Population: Patients with alveolar bone defects requiring regeneration, including those undergoing implant placement or other oral rehabilitations.
- Intervention: Use of synthetic biomaterials (e.g., hydroxyapatite, β-tricalcium phosphate, bioglass) for guided bone regeneration or ridge augmentation.
- Comparison: Autologous, allogenic, or xenogenic grafts, other regenerative techniques, or no treatment.
- Outcomes: Primary outcomes included bone volume gain, residual biomaterial, implant stability, infection rate, and complications. Secondary outcomes included histological integration and implant success.

Information Sources and Search Strategy:

A comprehensive electronic search was conducted across PubMed, MEDLINE, Embase (via Ovid), and Google Scholar up to March 2025, without time restrictions. The last search was performed on 15 March 2025. The search strategy was primarily executed and optimized within PubMed and Google Scholar due to their comprehensive coverage and efficiency in indexing relevant biomedical literature.

The initial search strategy included articles published in English, Italian, or Spanish. Nonetheless, all records that met the eligibility criteria for final inclusion were reported in English, resulting the ultimate analysis being limited to English-language studies.

The search combined controlled vocabulary (MeSH) and free-text terms using Boolean operators as follows:

*(“bone regeneration” OR “guided bone regeneration” OR “dental bone graft”) AND (“synthetic biomaterials” OR “hydroxyapatite” OR “beta-tricalcium phosphate” OR “bioceramics” OR “bioactive glass”) AND (“dental implants” OR “oral surgery”).*

Manual searches of reference lists and citation tracking were also performed to identify additional eligible studies.

Study Selection:

Two independent reviewers screened all titles and abstracts for relevance. Full-text articles of potentially eligible studies were retrieved and assessed according to the eligibility criteria. Discrepancies were resolved by consensus or consultation with a third reviewer.

All the retrieved references were imported into a reference management software (e.g., Microsoft Excel), and duplicates were automatically removed. The search was then complemented by manual screening of reference lists from included articles and relevant reviews. Animal studies, in vitro experiments, case reports, narrative reviews, and systematic reviews were excluded at this stage of screening. Conversely, papers dealing with human subjects and investigating the use of synthetic biomaterials for alveolar ridge maintenance and regeneration were included.

Full texts of potentially eligible studies were reviewed to determine final inclusion. To maintain methodological rigor and ensure the inclusion of original data, review articles were excluded. Their secondary nature and lack of primary outcomes rendered them unsuitable for the objectives of this systematic review. The same rationale applied to case reports, which typically lack comprehensive data and are not designed to support systematic analysis.

Reasons for exclusion were systematically documented and are summarized in the PRISMA flowchart (Supplementary Table S1).

Data Collection Process:

Data extraction was independently performed by two reviewers using a standardized pre-piloted form. Extracted data included: study design, sample size, demographic and clinical characteristics, intervention and comparator details, biomaterial composition, follow-up duration, and quantitative outcomes (bone gain, residual biomaterial, implant success, complications). When required, corresponding authors were contacted to obtain missing or unclear data. Data extraction was verified for consistency by a third reviewer.

Quality Assessment and Risk of Bias:

The methodological quality of RCTs was evaluated using the Cochrane Risk of Bias (RoB 2.0) tool, and non-randomized studies were appraised using the ROBINS-I instrument (Cochrane Collaboration, London, UK) [16]. Each domain was graded as low, unclear, or high risk of bias according to explicit methodological details (random sequence generation, allocation concealment, blinding, and outcome reporting). The overall certainty of evidence was rated using the GRADE (Grading of Recommendations Assessment, Development and Evaluation) framework, allowing classification as high, moderate, low, or very low certainty.

Two reviewers assessed the risk of bias independently, with disagreements resolved by discussion.

Data Synthesis:

Due to the marked heterogeneity of study designs, outcome measures, and biomaterial formulations, quantitative synthesis (meta-analysis) was not feasible. Instead, data were synthesized descriptively, highlighting comparative trends, effect consistency, and the relative performance of different biomaterial categories.

## 3. Results

**Figure 1:**
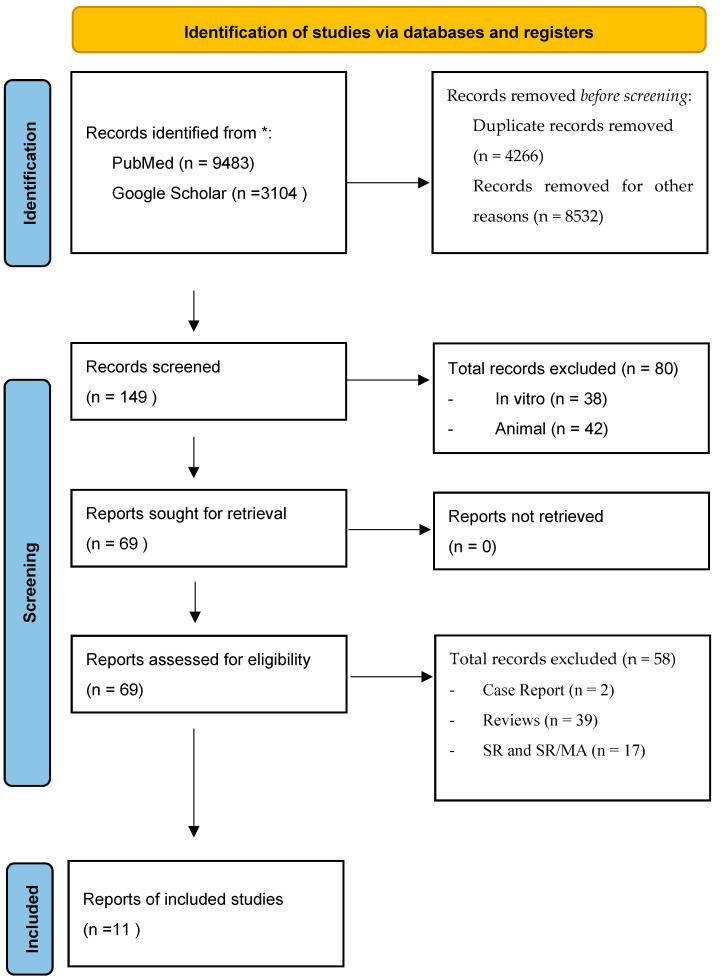
PRISMA 2020 flow diagram for new systematic reviews [18]. * reporting the articles selection process.

Initially, our literature search yielded 12,947 records. The selection of studies was carried out in accordance with the PRISMA (Preferred Reporting Items for Systematic Reviews and Meta-Analyses) guidelines [18] (Figure 1). Primarily, any duplicate records were removed.

One study was also excluded due to readability issues. Subsequently, we conducted a thorough screening of the titles, abstracts, and full texts of potentially eligible studies. As a result, studies conducted in vitro or involving animal subjects were excluded. This rigorous process resulted in a selection of 69 studies on human subjects from which, in accordance with screening criteria and methods, unsuitable or improper studies were removed resulting in a final collection of 11 studies (Table 1).

Data Synthesis: Despite including RCTs and non-randomized comparative studies, the high heterogeneity in study design, interventions, and reported outcomes did not allow us to perform a meta-analysis.

The included studies differed markedly in several methodological and analytical aspects, which were consistent with the respective study objectives and, at the same time, provided a broader range of parameters for the evaluation of the same material or treatment. Many studies focused on the collection of purely biological data, while others assessed more quantitative or instrumental parameters.

Data are documented in Table 1 and Table 2.

At the end of the process, 11 studies met the inclusion criteria, as reported in Figure 1: Tomas MK et al. [17]; Wang YF et al. [19]; Prins HjS et al. [20]; Gjerde CM et al. [21]; Sarment DP et al. [22]; Nagaveni NBP et al. [23]; Gupta AKA et al. [24]; Canuto RAP et al. [25]; Troedhan AS et al. [26]; Sun Y et al. [27]; and Wei LS et al. [28].

Detailed information on the included studies is reported in Table 1.

**Figure 2:**
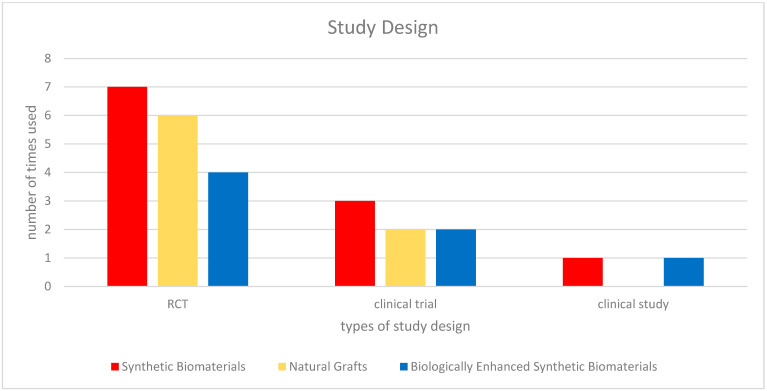
Overall study designs. *If different types of materials were used within a single study, they were considered in multiple groups*.

**Figure 3:**
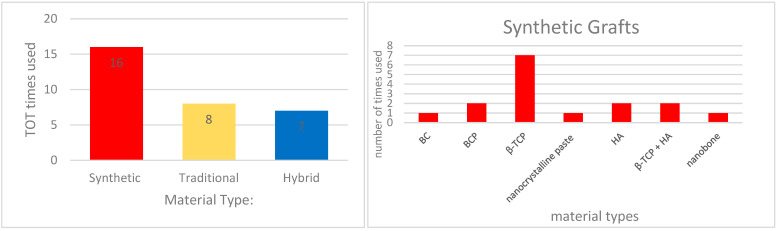

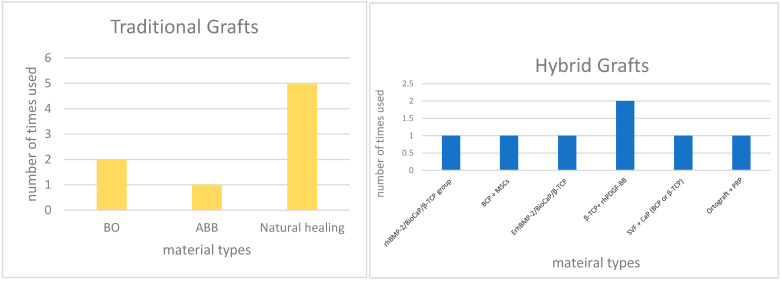
Material types. *If different types of materials were used within a single study, they were considered in multiple groups*.

**Figure 4:**
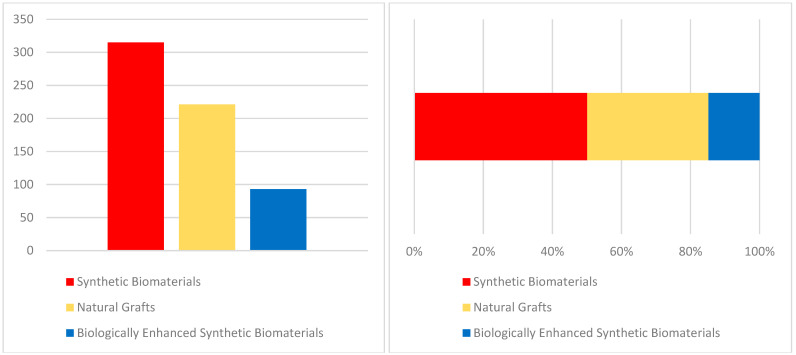
Sample Size. *If different types of materials were used within a single study, they were considered in multiple groups*.

**Figure 5:**
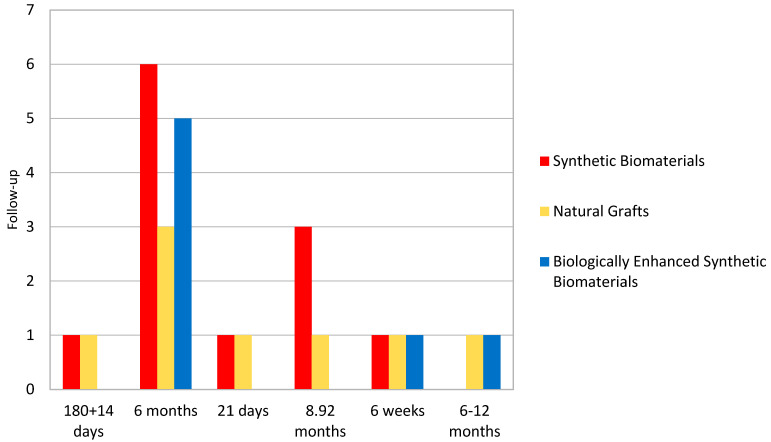
Follow-up Time. *If different types of materials were used within a single study, they were considered in multiple groups*.

**Figure 6:**
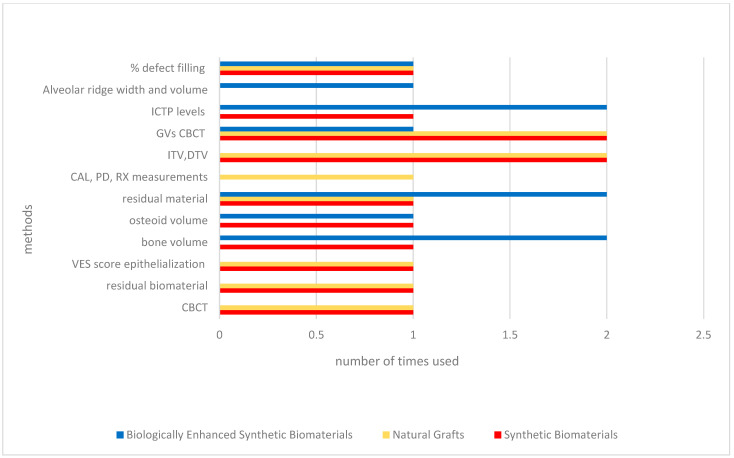
Methods. *If different types of materials were used within a single study, they were considered in multiple groups*.

**Figure 7:**
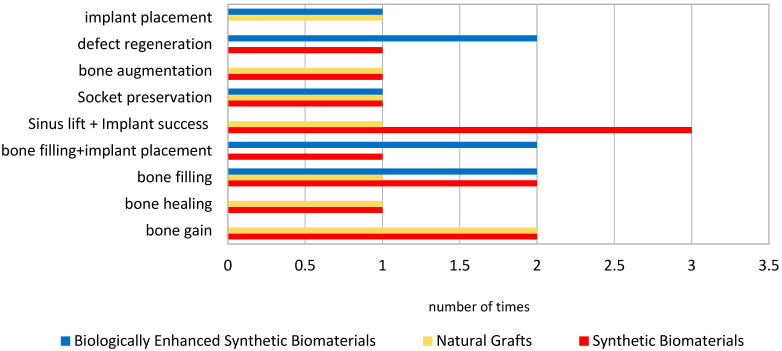
Clinical Outcomes. *If different types of materials were used within a single study, they were considered in multiple groups*.

Randomized and controlled clinical trials investigating synthetic biomaterials for osseous restoration were analyzed. The studies collectively addressed implant placement in sites with compromised bone volume, comparing synthetic grafts with autologous, allogenic, and xenogenic substitutes (Figure 2, Figure 3, Figure 4, Figure 5, Figure 6 and Figure 7). To provide a comprehensive overview of the included studies, key parameters such as study design, sample size, and follow-up duration were systematically evaluated (Figure 2, Figure 3, Figure 4 and Figure 5). Across the included investigations, outcome measures primarily comprised quantitative assessments of bone formation, biomaterial resorption, and implant stability, supported by histological and radiographic evidence (Figure 6 and Figure 7).

### 3.1. Bone Regeneration with Synthetic Biomaterials

Synthetic substitutes demonstrated consistent performance in promoting bone augmentation compared to conventional xenografts (Table 3). In the randomized trial by Wang YF et al. [19], a novel bioceramic (BC) exhibited slightly greater horizontal bone thickness preservation than a bovine-derived xenograft (BO), both immediately postoperatively and after 180 ± 14 days, confirming stable dimensional outcomes (mean HT: 5.775 ± 1.345 mm vs. 5.273 ± 1.285 mm). Tomas MK et al. [17] reported comparable results between injectable biphasic calcium phosphate (BCP) and anorganic bovine bone (ABB), noting lower residual biomaterial for BCP (28.61 ± 11.38%) versus ABB (31.72 ± 15.52%), although differences were not statistically significant (*p* > 0.05).

The clinical trial by Canuto RA et al. [25] assessed nanocrystalline hydroxyapatite (Ostim^®^) as a post-extractive filler. Healing was characterized by early epithelialization (day 7) and upregulation of VEGF and IL-10, supporting angiogenesis and anti-inflammatory activity. Despite transient postoperative pain (VAS peak on day 2 due to IL-1β increase), the Ostim^®^ group achieved faster resolution and superior osteogenic signaling, suggesting enhanced biological integration.

Gupta AKA et al. [24] extended the evaluation of hydroxyapatite/β-TCP to periodontal defects, demonstrating greater clinical and radiographic improvement than open flap debridement (OFD). After a six-month period, the treated group showed a 44.7% reduction in probing depth and 41.1% gain in attachment level, with bone fill reaching 42.6% versus 20.0% in controls (*p* < 0.05).

### 3.2. Comparative Efficacy of Natural and Synthetic Grafts

When compared with traditional grafts, synthetic biomaterials achieved equivalent clinical performance (Table 4). In Wang YF et al. [19], although buccal bone thickness was initially greater in the xenograft group (3.633 ± 1.105 mm vs. 3.421 ± 1.227 mm), long-term measurements favored the synthetic material (2.476 ± 1.141 mm vs. 2.042 ± 1.097 mm). Similarly, Tomas MK et al. [17] found comparable new bone formation (41.73 ± 13.99% for ABB—biphasic calcium phosphate vs. 39.91 ± 8.40% for BCP—anorganic bovine bone) and soft tissue proportions, indicating no significant differences (*p* > 0.05).

Troedhan AS et al. [26] reported superior implant stability following sinus lift procedures using Bio-Oss, with Drill Torque Value (DTV) and Implant Torque Value (ITV) significantly higher than natural subantral bone (12.7 vs. 10.2 Ncm; 26.2 vs. 22.2 Ncm, respectively; *p* < 0.05).

In the study by Gjerde CM et al. [21], autologous mesenchymal stromal cells (MSCs) combined with BCP achieved a mean alveolar ridge width increase of 4.05 mm and volume gain of 887.2 ± 365.0 mm^3^, confirming a synergistic effect.

### 3.3. Variability Among Synthetic Biomaterials

The choice of synthetic material strongly influenced clinical outcomes (Table 5). Troedhan AS et al. [26] demonstrated significant intermaterial differences in DTV and ITV among easy-graft CRYSTAL, easy-graft CLASSIC, NanoBone, Bio-Oss, and natural bone (*p* < 0.001). The easy-graft CRYSTAL group recorded the highest DTV (23.8 ± 3.1 Ncm) and ITV (56.6 ± 3.4 Ncm), suggesting that materials forming rigid, cohesive scaffolds yield improved primary stability. Conversely, NanoBone, despite its hydroxyapatite content, performed less favorably due to its elastic, mobile consistency under sinus pressure, emphasizing the mechanical relevance of graft consolidation (unlike easy-graft CLASSIC and CRYSTAL, which rapidly harden into a stable block-like graft upon blood contact).

### 3.4. Biologically Enhanced Synthetic Biomaterials

Several studies evaluated hybrid approaches combining synthetic scaffolds with bioactive molecules (Table 6). Sun Y et al. [27] showed that rhBMP-2/BioCaP/β-TCP induced significantly higher new bone area (21.18% ± 7.62%) and lower residual material (10.04% ± 4.57%) than β-TCP alone (new bone: 13.44% ± 6.03%; residual material: 20.60% ± 9.54%) (*p* < 0.05).

Wei LS et al. [28] confirmed similar advantages with ErhBMP-2/BioCaP/β-TCP, which achieved reduced residual material (10.90% ± 4.04%) and greater bone volume density (7.72% ± 6.01%) relative to β-TCP (residual material: 15.73% ± 4.52%; bone volume: 2.96% ± 2.23%) (*p* < 0.05).

Sarment DP et al. [22] observed increased ICTP release in rhPDGF-treated groups, consistent with stimulated bone turnover, though not statistically significant (*p* > 0.05). Prins HJ et al. [20] found that incorporating stromal vascular fraction (SVF) into β-TCP or BCP improved micro-CT bone volume (19.5% ± 3.8% vs. 13.7% ± 4.4%; *p* = 0.03), even without radiological differences in height or graft volume.

In pediatric cystectomy patients, Nagaveni NB et al. [23] demonstrated a significantly greater defect fill using PRP combined with Ortograft (94% vs. 47%; *p* < 0.001), supporting the role of platelet-derived factors in accelerating bone maturation.

### 3.5. Limitations and Gaps

Although findings consistently favored synthetic biomaterials, several methodological constraints were noted (Table 7). Troedhan AS et al. [26] emphasized that DTV alone inadequately reflects bone quality, advocating ITV as a more reliable intraoperative metric. Gjerde CM et al. [21] identified logistical limitations in cell culture timing, while Prins HJ et al. [20] reported minor discrepancies between histomorphometric and micro-CT analyses. Sun Y et al. [27] noted that CBCT grayscale variations may not fully capture tissue mineralization, and Tomas MK et al. [17] highlighted inconsistencies in *p*-value reporting (e.g., *p* < 0.05 misclassified as non-significant).

Collectively, these issues underline the need for standardized protocols, uniform statistical reporting, and broader comparative analyses to strengthen the evidence base for synthetic biomaterials in alveolar bone regeneration.

## 4. Discussion

The present review evaluated the clinical performance of synthetic biomaterials in alveolar bone regeneration, including biphasic calcium phosphate (BCP), β-tricalcium phosphate (β-TCP), nanocrystalline hydroxyapatite, and bioactive glass composites. Overall, the evidence supports their capacity to achieve adequate bone volume preservation, histological integration, and implant stability, often comparable to traditional autografts, allografts, and xenografts. However, a more critical comparison among these classes of synthetic biomaterials highlights distinct strengths and limitations: for instance, β-TCP exhibits faster resorption but limited volume stability, while BCP provides a more balanced degradation profile; conversely, bioactive glass demonstrates superior osteostimulation but poses handling challenges in particulate form. However, a deeper analysis reveals that their clinical effectiveness is influenced not only by chemical composition but also by microstructural and biological factors that govern osteoconductivity and remodeling. These factors include pore size, interconnectivity, and surface chemistry, which modulate protein adsorption and cellular adhesion, ultimately determining the osteoconductive and osteoinductive potential of the scaffold.

Several studies within this review found minimal or no statistically significant differences between synthetic biomaterials and xenografts. Tomas et al. [17] reported equivalent outcomes between injectable BCP and anorganic bovine bone (Bio-Oss^®^), although numerical trends favored synthetics in residual material resorption. Similarly, Wang et al. [19] demonstrated that a novel bioceramic provided slightly superior long-term horizontal bone preservation compared with bovine xenograft. These findings suggest that synthetic materials, when adequately engineered, can reproduce the biological environment of xenografts, offering comparable outcomes with reduced variability and no risk of disease transmission. These findings are consistent with the meta-analysis by Klijn et al. [14], which concluded that selected synthetic substitutes can achieve histomorphometric results comparable to autologous bone in sinus floor augmentation. Collectively, these observations suggest that properly engineered synthetic scaffolds can replicate the osteoconductive environment of natural bone matrices when adequate porosity, interconnectivity, and surface topography are achieved. Nonetheless, it remains essential to recognize that the osteoinductive potential of synthetic biomaterials is more limited than that of autografts, requiring biological enhancement to achieve comparable cellular recruitment and differentiation.

Mechanistically, the performance of synthetic biomaterials depends on critical parameters such as porosity, surface energy, and degradation kinetics. Highly porous and interconnected structures facilitate vascular and cellular infiltration, accelerating osteoid deposition and bone maturation. However, excessive resorption rates may compromise scaffold stability before new bone formation occurs, while overly dense materials can hinder cell migration and nutrient diffusion. Thus, balancing resorption with osteogenesis remains central to optimizing regenerative outcomes. These aspects are insufficiently addressed in most included clinical studies, which focus primarily on histological or radiographic endpoints rather than correlating material properties with biological behavior. Future research should therefore integrate physico-chemical characterization with histomorphometric outcomes to better elucidate the biological mechanisms underlying material performance.

The incorporation of bioactive molecules into synthetic scaffolds emerged as a promising direction to overcome such limitations. Sun Y et al. [27] and Wei et al. [28] showed that β-TCP combined with rhBMP-2 significantly increased new bone formation and reduced residual biomaterial compared with β-TCP alone. Similar enhancements were observed when β-TCP was associated with platelet-rich plasma (PRP) or rhPDGF-BB, as demonstrated by Nagaveni et al. [23] and Sarment DP et al. [22], confirming the potential of osteoinductive enrichment to modulate cell signaling pathways and accelerate regeneration. These results are in line with Miron & Zhang [12], who emphasized that the osteoinductive potential of synthetic scaffolds depends on both the controlled release of growth factors and the maintenance of a three-dimensional environment conducive to cell attachment and differentiation. In this context, emerging technologies such as 3D-printed scaffolds, bioactive surface coatings, and hybrid composites integrating growth factors or stem cells represent a new frontier in biomaterial design, potentially bridging the gap between purely osteoconductive and truly osteoinductive systems [29,30,31].

Nevertheless, the clinical performance of synthetic substitutes varied markedly among materials with similar chemical compositions [32,33,34,35]. Troedhan et al. [26] identified significant differences in implant torque values between bioceramics of comparable composition, underscoring that microstructural architecture, surface roughness, and cohesive behavior under load are decisive for implant stability. The findings corroborate the conclusions of several other evidences regarding the need and the outcomes of bone augmentation in implant dentistry [36,37,38,39]. As further confirmation, Troeltzsch et al. [40] observed that implant success and bone regeneration outcomes are influenced as much by the material’s physical handling and mechanical resilience as by its chemistry. From a clinical standpoint, ease of manipulation, intraoperative stability, and cost-effectiveness are equally crucial for translating laboratory performance into predictable surgical outcomes, aspects that are often underreported in clinical trials. This reinforces the need to integrate material science parameters—such as elastic modulus, crystallinity, and surface energy—into the clinical evaluation of bone grafts.

Despite encouraging outcomes, heterogeneity across study designs, control groups, and follow-up durations limits comparability. The absence of standardized outcome measures—ranging from histomorphometric percentages to torque-based metrics—precluded quantitative synthesis. Most included trials involved small sample sizes and short-term follow-ups, issues that mirror those identified by Esposito et al. [41] in the Cochrane review on bone augmentation. Moreover, patient-centered outcomes, such as postoperative function, implant longevity, and esthetic satisfaction, were rarely reported. Chan et al. [42], Lin, Y.; [43], Zhou et al. [44] similarly emphasized that the long-term clinical relevance of bone regeneration strategies cannot be fully assessed without incorporating functional and quality-of-life indicators. These limitations highlight the need for well-designed randomized controlled trials with larger cohorts, standardized histomorphometric analysis, and long-term follow-up to produce more reliable evidence.

From a translational perspective, synthetic biomaterials offer notable advantages, including unlimited availability, absence of donor-site morbidity, and the potential for molecular customization through incorporation of peptides, ions, or stem-cell derivatives. Their performance, however, must be interpreted in light of defect-specific biomechanics, vascular supply, and surgical technique [45]. The selection of a biomaterial should therefore be guided by a tailored approach, balancing scaffold stability and degradation kinetics with the biological demands of the recipient site. Clinicians should also consider economic aspects and ease of handling when selecting materials for clinical use, as these practical parameters substantially affect treatment feasibility and patient outcomes.

In summary, the current evidence indicates that synthetic biomaterials represent viable alternatives to traditional grafts in alveolar bone regeneration. However, their use should be framed within a critical understanding of their biological and mechanical limitations compared to autologous bone. Future studies should aim to establish standardized characterization methods linking material physico-chemical parameters with biological outcomes, integrate advanced imaging and computational modeling to predict resorption dynamics, and adopt longer follow-up designs to assess true clinical performance. Furthermore, the inclusion of emerging biomaterial technologies such as 3D printing, bioactive coatings, and growth factor-enriched hybrids could pave the way toward next-generation scaffolds capable of personalized and predictable regeneration. Such integration of materials science and clinical research will be essential to move beyond descriptive findings and toward evidence-based optimization of synthetic grafts for predictable and durable bone regeneration.

## 5. Conclusions

Synthetic biomaterials—especially biphasic calcium phosphates, β-tricalcium phosphates, and nanostructured hydroxyapatite—are effective and practical alternatives to autologous and xenogenic grafts for alveolar bone regeneration. When correctly indicated, they ensure comparable bone preservation, integration, and implant stability, while avoiding donor-site morbidity.

Their performance depends on both composition and microstructure, influencing osteoconductivity and resorption. Synthetic materials are particularly useful in small to moderate defects, sinus lifts, and ridge augmentations where mechanical load is limited. In larger or complex defects, reinforcement or bioactive enhancement (e.g., rhBMP-2, rhPDGF-BB) may be required to optimize outcomes.

Although current data confirm their clinical reliability, heterogeneity among studies and limited follow-ups prevent firm conclusions about material superiority. Future research should focus on standardized, long-term randomized trials evaluating specific endpoints—bone integration, quantitative gain, and implant stability. Including patient-reported outcomes will enhance the clinical relevance of future findings.

Ultimately, a clearer understanding of material behavior will allow clinicians to identify when synthetic biomaterials represent the best regenerative option and integrate them effectively into daily dental practice.

## Figures and Tables

**Table 1:** Characteristics of the included studies.

Author	Type of Intervention	Comparison/Control	Outcomes Measured	Conclusions
Tomas MK et al. [17]	Effectiveness comparison among different materials	ABB (Bio-oss^®^) and Injectable BCP (maxresorb^®^ inject—16.5% biphasic granules and 83.5% nano-HA gel)	Differences between newly formed bone, residual biomaterial and soft tissue	No significant difference found between the groups
Wang YF et al. [19]	GBR during implant placement	Novel bioceramic (BC) versus a control xenograft (BO)	CBCT images at T1 and T2 measuring horizontal buccal bone thicknesss and width change Soft tissue gealing status	Novel BC material is safe and effective, efficacy comparable to xenograft bone
Prins HjS et al. [20]	6 bilaterally treated 4 unilateral	β-TCP and BCP ceramics combined with SVF versus β-TCP and BCP without SVF	Micro-CT and quantitative histomorphometric evaluations on both unilateral and bilaterally treated patients	In study biopsies (with SVF) bone and osteoid % were higher Pared analysis on bilaterally treated patients revealed higher bone and osteoid % when treated adding SVF
Gjerde CM et al. [21]	Bone regeneration using MSCs	BCP granules with MSCs versus BCP alone	Average bone healing, increased bone width and volume were statistically relevant	MSCs induce significant formation of new bone
Sarment DP et al. [22]	Periodontal regenerative surgery	rhPDGF (+0.3 mg/mL) with β-TCP, rhPDGF (+1.0 mg/mL) with β-TCP, versus β-TCP alone	Evaluate release of the ICTP into periodontal wound fluid	rhPDGF-BB has a direct effect on ICTP released from the wound
Nagaveni NBP et al. [23]	Bone regeneration after cystectomy	adding PRP to a bone graft in the versus without PRP	After 6 months, defect bone fills show 94% filling in test group and 47% in the control group	PRP application improves bone height regeneration
Gupta AKA et al. [24]	Periodontal regenerative surgery	β-TCP versus open flap debridement only	Probing pocket depth, Clinical attachment level, Radiological measurements	β-TCP reduces probing depth almost by 75% and gain clinical attachment
Canuto RAP et al. [25]	Bone regeneration for post-extractive socket treatment using the graft materials without elevation of full-thickness flaps	Ostim^®^-filled socket vs. without Ostim^®^	Clinical control (VAS score) and biological parameters (synthesis of pro-osteogenic factors, as BMP-4, BMP-7, alkaline phosphatase, osteocalcin)	Ostim^®^ increases the production of positive molecules for socket healing but causes greater pain at day 1
Troedhan AS et al. [26]	Bone regeneration and implant insertion	Bio-Oss^®^, Nano-Bone, easy-graft CLASSIC, easy-graft CRYSTAL in alternative to natural subantral bone	Drill Torque Value (DTV) and Implant-insertion Torque Value (ITV)	Statistical supremacy of easy-graft CRYSTAL
Sun Y et al. [27]	Socket preservation and bone formation	rhBMP-2/BioCaP/beta-TCP, beta-TCP, natural healing	Radiographic evaluation (change in GVs in CBCT scans) and Histomorphologic (% of new bone, residual material areas, unmineralized tissue area)	hBMP-2/BioCaP/beta-TCP is a promising substitute
Wei LS et al. [28]	Tooth-extraction-healing model before implant placement surgery	ErhBMP-2/BioCaP/beta-TCP, beta-TCP, natural healing	% of new bone, residual material areas, unmineralized tissue area	ErhBMP-2/BioCaP/β-TCP is a promising substitute

**Table 2:** Reports the assessment of the risk of bias.

	Risk of Bias Arising from the Randomization Process	Effect of Assignment to Intervention	Risk of Bias Due to Missing Outcome Data	Risk of Bias in Measurement of the Outcome	Risk of Bias in Selection of the Reported Result	Overall Risk-of-Bias Judgment
Tomas MK et al. (2023) [17]						Some Concerns
Wang YF et al. (2025) [19]						Low Risk
Prins HJS et al. (2016) [20]						High Risk of bias
Gjerde CM et al. (2018) [21]						Some Concerns
Sarment DP et al. (2006) [22]						Some Concerns
Nagaveni NBP et al. (2010) [23]						Some Concerns
Gupta AKA et al. (2022) [24]						Some Concerns
Canuto RAP et al. (2013) [25]						Some Concerns
Troedhan AS et al. (2014) [26]						Low Risk of bias
Sun Y et al. (2023) [27]						Some Concerns
Wei LS et al. (2025) [28]						Low Risk of bias

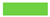
 green = low risk of bias; 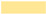 yellow = some concerns; 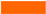 red = high risk of bias. A study was categorized as high-risk or having some concerns if it met these criteria in at least one domain.

**Table 3:** Overview of the main results discussed in the text.

	*Author*	*Subjects*	*Measurements*	*Results*
*Section 3.1*	Wang YF et al. [19]	novel bioceramic (BC), control xenograft (BO) for GBR	horizontal bone thickness (HT) at T1 (post-operative) and T2 (180 ± 14 days postoperatively)	HT at T1 BC: 6.053 ± 1.262 mm BO: at 5.907 ± 1.269 mm. HT at T2 BC: 5.775 ± 1.345 mm BO: 5.273 ± 1.285 mm
	Tomas MK et al. [17]	ABB (Bio-oss^®^), Injectable BCP (maxresorb^®^ inject)	% of residual biomaterial	BCP: 28.61 ± 11.38% ABB: 31.72 ± 15.52%
	Canuto RAP et al. [25]	Ostim pastes	VAS score Pro-osteogenic factors augmentation	VES: 5 at day 2 Increase VEGF, IL-10, IL-1β expression
	Gupta AKA et al. [24]	biphasic hydroxyapatite BHA and β-tricalcium phosphate, OFD	probing depth clinical attachment % bone filling in RX	probing depth reduction BHA—β-TCP: 44.70% OFD: 31.37% clinical attachment gain BHA—β-TCP: 41.07% OFD: 29.87% % bone filling BHA—β-TCP: 42.57% OFD: 20.04%

**Table 4:** Overview of the main results discussed in the text.

	*Author*	*Subjects*	*Measurements*	*Results*
*Section 3.2*	Wang YF et al. [19]	novel bioceramic (BC), control xenograft (BO) for GBR	buccal bone thickness T1 (post-operative) and T2 (180 ± 14 days postoperatively) % post-operative heling status at T1 (14 days postoperative) and T2 (30 days postoperative)	buccal bone thickness at T1 BO: 3.633 ± 1.105 mm BC: 3.421 ± 1.227 mm buccal bone thickness at T2 BO: 2.042 ± 1.097 mm BC: 2.476 ± 1.141 mm No inflammation at T1 BO: 90.79% BC: 96.05% No inflammation at T2 BO: 98.68% BC: 97.37%
	Tomas MK et al. [17]	ABB (Bio-oss^®^), Injectable BCP (maxresorb^®^ inject)	% newly formed bone (NB) % soft tissue (ST)	new bone ABB: 41.73 ± 13.99% ABB: 39.91 ± 8.40% soft tissue ABB: 26.54 ± 7.25% BCP: 31.49 ± 11.09%)
	Troedhan AS et al. [26]	Bio-Oss^®^, natural bone (NB)	DTV and ITV (Ncm)	DTV mean value BO: 12.7 Ncm NB: 10,2 Ncm ITV mean value BO: 26.2 Ncm NB: 22.2 Ncm
	Gjerde CM et al. [21]	BCP/MSCs	width and volume of alveolar ridge	average bone width increase 4.05 mm average bone volume increase 887.23 ± 365.01 mm3

**Table 5:** Overview of the main results discussed in the text.

	*Author*	*Subjects*	*Measurements*	*Results*
*Section 3.3*	Troedhan AS et al. [26]	easy-graft CRYSTAL, easy-graft CLASSIC, NanoBone, Bio-Oss, natural healing (NB)	DTV and ITV (Ncm) chemical composition and behavior	DTV mean value NB: 10.2 Ncm Bio-Oss: 12.7 Ncm NanoBone: 17.5 Ncm easy-graft CLASSIC: 20.3 Ncm Easy-graft CRYSTAL: 23.8 Ncm ITV mean value NB: 22.2 Ncm Bio-Oss: 26.2 Ncm NanoBone: 33.3 Ncm easy-graft CLASSIC: 45.9 Ncm easy-graft CRYSTAL: 56.6 Ncm chemical composition Bio-Oss: anorganic bovine bone easy-graft CRYSTAL: 40% beta-tricalcium phosphate (β-TCP) and 60% hydroxyapatite (HA), coated by a 10-micrometer layer of polylactic-co-glycolic acid (PLGA). easy-graft CLASSIC: pure β-TCP particles. NanoBone: Nanocrystalline HA embedded in a SiO2 matrix

**Table 6:** Overview of the main results discussed in the text.

	*Author*	*Subjects*	*Measurements*	*Results*
*Section 3.4*	Sun Y et al. [27]	rhBMP-2/BioCaP/β-TCP, β-TCP, natural healing	% newly formed bone (NB) % residual material (RM) % unmineralized tissue area (UT)	NB rhBMP-2/BioCaP/β-TCP: 21.18% ± 7.62% β-TCP: 13.44% ± 6.03% natural healing: 9.49% ± 0.08% RM rhBMP-2/BioCaP/β-TCP: 10.04% ± 4.57% β-TCP: 20.60% ± 9.54% UT rhBMP-2/BioCaP/β-TCP: 68.78% ± 7.67% β-TCP: 65.96% ± 12.64% natural healing: 90.38% ± 7.5%
	WEI LS et al. [28]	ErBMP-2/BioCaP/β-TCP, β-TCP, natural healing	% newly formed bone (NB) % residual material (RM) % unmineralized tissue area (UT)	NB ErhBMP-2/BioCaP/β-TCP: 7.72% ± 6.01% β-TCP: 2.96% ± 2.23% natural healing: 8.37% ± 6.31% RM ErhBMP-2/BioCaP/β-TCP: 10.90% ± 4.04% β-TCP: 15.73% ± 4.52% UT ErhBMP-2/BioCaP/β-TCP: 81.38% ± 4.81% β-TCP: 81.32% ± 4.70% natural healing: 91.63% ± 6.31%
	Sarment DP et al. [22]	β-TCP, β-TCP + rhPDGF-BB 0.3 mg/mL β-TCP + rhPDGF-BB 1.0 mg/ml	release of ICTP (pg/site)	ICTP levels (overall AUC values) rhPDGF-BB 0.3 mg/mL: 641.7 ± 164.1 pg/10 s rhPDGF-BB 1.0 mg/mL: 672.8 ± 183.0 pg/10 s β-TCP: 437.5 ± 287.1
	Prins HjS et al. [20]	BCP, β-TCP, BCP/SVF β-TCP/SVF	mm vertical bone augmentation % bone volume % graft volume % bone volume proportion	bone augmentation β-TCP: 10.261.5 mm β-TCP/SVF: 9.9 ± 1.3 mm BCP: 12.4 ± 1.6 mm BCP/SVF: 12.1 ± 1.6 mm bone volume SVF sides: 19.5% ± 3.8% control sides: 13.7% ± 4.4% graft volume SVF sides: 10.5% ± 3.6% control sides: 14.0% ± 3.6% bone volume proportion SVF sides: 15.2% ± 4.7% control sides: 13.3% ± 3.0%
	Nagaveni NBP et al. [23]	Ortograft/PRP, Ortograft	% filling defect mm reduction defect	Ortograft/PRP: 9.5 ± 1.0 mm to 1.3 ± 2.1 mm (94% defect filled) Ortograft: reached 47% defect fill only at the 6th month

**Table 7:** Overview of the main results discussed in the text.

	*Author*	*Subjects*	*Measurements*	*Results*
*Section 3.5*	Troedhan AS et al. [26]	DTV and ITV	Ncm	DTV: insufficient average differences (2–5 Ncm) between materials and bone ITV: highly significant differences 4 Ncm difference between native bone and Bio-Oss 34 Ncm in easy-graft CRYSTAL-augmented sites DTV and ITV specific to the Q2-implant
	Gjerde CM et al. [21]			critical number of cells or a specific cell-to-biomaterial ratio (BCP) seems to be a key factor influencing bone formation
	Prins HjS et al. [20]			minor discrepancies between the histomorphometric results and micro-CT findings regarding bone and graft volumes
	Sun Y et al. [27]			CBCT data may not be sufficient for a comprehensive analysis (revealed significant differences in grayscale value)
	Tomas MK et al. [17]			newly formed bone, residual biomaterial and soft tissue show *p* = 0.629, *p* = 0.485, *p* = 0.113, respectively, = *p*-value should be *p* > 0.05

## Data Availability

No new data were created or analyzed in this study. Data sharing is not applicable to this article.

## Supplementary Materials

The following supporting information can be downloaded at: https://www.mdpi.com/article/10.3390/ma18235328/s1, Table S1. PRISMA 2020 Checklist.
